# Socio-cultural perception of robot backchannels

**DOI:** 10.3389/frobt.2023.988042

**Published:** 2023-01-26

**Authors:** Olov Engwall, Ronald Cumbal, Ali Reza Majlesi

**Affiliations:** ^1^ Division of Speech, Music and Hearing, School of Electrical Engineering and Computer Science, KTH Royal Institute of Technology, Stockholm, Sweden; ^2^ Department of Education, Stockholm University, Stockholm, Sweden

**Keywords:** human-robot conversation, backchannel behavior, socio-cultural effects, robot-assisted language learning, multiparty interaction

## Abstract

**Introduction:** Backchannels, i.e., short interjections by an interlocutor to indicate attention, understanding or agreement regarding utterances by another conversation participant, are fundamental in human-human interaction. Lack of backchannels or if they have unexpected timing or formulation may influence the conversation negatively, as misinterpretations regarding attention, understanding or agreement may occur. However, several studies over the years have shown that there may be cultural differences in how backchannels are provided and perceived and that these differences may affect intercultural conversations. Culturally aware robots must hence be endowed with the capability to detect and adapt to the way these conversational markers are used across different cultures. Traditionally, culture has been defined in terms of nationality, but this is more and more considered to be a stereotypic simplification. We therefore investigate several socio-cultural factors, such as the participants’ gender, age, first language, extroversion and familiarity with robots, that may be relevant for the perception of backchannels.

**Methods:** We first cover existing research on cultural influence on backchannel formulation and perception in human-human interaction and on backchannel implementation in Human-Robot Interaction. We then present an experiment on second language spoken practice, in which we investigate how backchannels from the social robot Furhat influence interaction (investigated through speaking time ratios and ethnomethodology and multimodal conversation analysis) and impression of the robot (measured by post-session ratings). The experiment, made in a triad word game setting, is focused on if activity-adaptive robot backchannels may redistribute the participants’ speaking time ratio, and/or if the participants’ assessment of the robot is influenced by the backchannel strategy. The goal is to explore how robot backchannels should be adapted to different language learners to encourage their participation while being perceived as socio-culturally appropriate.

**Results:** We find that a strategy that displays more backchannels towards a less active speaker may substantially decrease the difference in speaking time between the two speakers, that different socio-cultural groups respond differently to the robot’s backchannel strategy and that they also perceive the robot differently after the session.

**Discussion:** We conclude that the robot may need different backchanneling strategies towards speakers from different socio-cultural groups in order to encourage them to speak and have a positive perception of the robot.

## 1 Introduction

Backchannel responses (Yngve, 1970), such as short verbal acknowledgements (e.g., “*uh-huh*”, “*mhm*”, “*yes*”) or non-verbal signals (e.g., head nods), are important in human conversations, as they indicate to the active speaker that the interlocutor is listening and following the line of reasoning, without having to resort to explicit requests for confirmation. Backchannels often come very natural in human-human conversations (Jefferson, 1984; Jefferson, 1993), as children from a young age learn this interactive strategy (Yamashita, 2017; Bodur et al., 2022) to signal either general attention (in which case they are provided at suitable opportunities given the speaker’s pauses or end of utterances) or receipt of specific information (in which case they follow directly after new, particularly important information) (Cutrone, 2010). Backchannels are provided without interrupting the turn of the speaker and may overlap with the speaker’s utterances (Jefferson, 1984; Jefferson, 1993; Heldner et al., 2013).

However, this seemingly natural interplay between the speaker and the listener’s backchannels occasionally breaks down because the listener and the speaker have differing views on how frequently or when backchannels should be provided and how they should be formulated. Too infrequent or too discrete backchannels may be taken as evidence that the listener is not paying attention. Too frequent or too intrusive backchannels may instead interrupt the speaker’s conversational flow and decrease enjoyment (Li et al., 2010) and satisfaction (Inden et al., 2013). Such problems may appear due to individual differences in interaction style, but they are substantially more frequent in intercultural conversations and when humans are interacting with conversational agents (e.g., voice assistants or social robots).

A large number of studies have demonstrated that distribution and perception of backchannels differ between different cultures and languages (White, 1989; Heinz, 2003; Cutrone, 2005; Mowlaei, 2017; Zellers, 2021) and that this may have consequences for intercultural conversation (White, 1989; Wan, 2018; Najim and Muhammad, 2020), unless the interlocutors adapt to the general backchannel strategy of the language spoken (Heinz, 2003; Cutrone, 2019). Other studies have shown that differences in the use and perception of backchannels are also related to gender (Mulac et al., 1998; Stubbe, 1998; Ueno, 2008), age (Yamashita, 2017; Kraaz and Bernaisch, 2022; Bodur et al., 2022) and familiarity with the speaker (Bodur et al., 2022).

Natural backchanneling is also challenging for conversational agents and robots and numerous studies (Gratch et al., 2007; Al Moubayed et al., 2009; Poppe et al., 2011; Inden et al., 2013; Hussain et al., 2019; Sebo et al., 2020; Adiba et al., 2021; Blomsma et al., 2022; Murray et al., 2022) have been devoted to endowing social robots and agents with a human-like and/or adequate backchannel strategy. It has been shown that the robot’s backchanneling affects how human subjects perceive it (Gratch et al., 2007; Blomsma et al., 2022) and it is thus a key factor for successful spoken human-robot interaction (HRI).

In this study we investigate the combined effect of intercultural conversation and HRI on the reaction to and perception of robot backchannels to investigate the research question: *Do socio-cultural factors*—*specifically gender*, *age*, *extroversion level and familiarity with robots*—*influence how L1 and L2 speakers respond to robot backchannels aimed at balancing the speaking activity in multiparty interaction?*

We first cover previous work on backchannels in interactions between humans (Section 2.1) and between humans and robots (Section 2.2). We then consider findings on how culture affects backchannels (Section 2.3), and discuss an expanded definition of culture, which relates not only to nationality or first language (as in the traditional view of cultural membership), but also to other socio-cultural factors that can affect backchannels (Section 2.4). How such socio-cultural factors affect the responses and perception of robot backchannels are then investigated experimentally in a robot-led triad spoken word game aimed at spoken practice for second language learners of Swedish (Section 3). The distribution of backchannels from a Furhat robot is modified in the experiment to attempt balancing the spoken interaction from the two human participants. In the analysis (Section 4), we specifically focus on how the subject’s socio-cultural background influence how they respond to and perceive the robot backchannels.

## 2 Previous work

Since the term backchannels was coined by Yngve (1970), this conversation feature has been the focus of a very large number of studies and it is therefore not possible to provide an exhaustive review of previous research. Instead, a selection of work that is most relevant for the present work is summarised, in particular regarding: formulation, frequency and timing of backchannels in human-human and human-robot conversations and how socio-cultural factors influence how backchannels are produced and interpreted.

### 2.1 Backchannels in human-human conversation

Backchannels may be described based on their functions, as displays of understanding, agreement, emphatic support, emotion, encouragement to continue or request for information or confirmation (Cutrone, 2010). The function may be either positive acknowledgement—verbal (e.g., *yes*, *mhm*) and visual (e.g., head nods and smiles)—or negative displays of non-understanding or disagreement—verbal (e.g., *hmm*…, *mhm?*) and visual (e.g., eyebrow frowning, raised eyebrows, pursed mouth).

They may also be categorised according to their form, both in terms of modality and lexicality. Traditionally, only vocal backchannels were considered, but more recent research is often including visual backchannels, separately or together with vocal backchannels. The vocal backchannels may be divided into if they are non-lexical (e.g., *mhm*) or lexical (e.g., *yes*) and if they are simple (mono- or bisyllabic expressions), double (repeated lexical form, e.g., *yeah-yeah*) or complex (combinations of different backchannel types) (Knight, 2009). For the present work, in which we strive to implement backchannels from a social robot and investigate how these are received by interlocutors of different background, the most important aspects are Formulation, Frequency and Timing of backchannels.

#### 2.1.1 Formulation of backchannels

Backchannels may be formulated in a great variety of ways, but it is often the case that a small number of backchannels dominate. For example, Wong and Peters (2007) showed that simple backchannels are the most common and White (1989) found that the five most common backchannels represented 76% of all occurrences (*mmhm* 43%; *yeah* 19%, *uh-huh* 18%, *oh* 14% and *hm* 6%). These backchannels, which can be used generically, are hence the attentive listener’s bread and butter. However, Tolins and Fox Tree (2014) demonstrated that generic (typically signals of understanding and attentiveness) and context-specific (e.g., emphatic support or display of emotion) influenced a storyteller differently, so that generic backchannels lead to discourse-new information, whereas context-specific lead to elaboration of previous information. For our experiment presented below, this hence indicates that the formulation of the robot’s backchannels may well have an impact on if and how the speakers continue.

#### 2.1.2 Frequency of backchannels

How frequently backchannels are uttered will naturally depend on the topic of the conversation, the speaker’s offers of backchannel opportunities and the individual listener, depending on factors such as degree of extroversion or socio-cultural traits (see further Sections 2.3, 2.4). Estimates regarding frequency of backchannels differ greatly between studies and languages: Gardner (2001) reports a rate of up to 3.33 backchannels per minute in English, whereas calculations of frequency from the figures reported by Heinz (2003) give an average of 8.9 and 6.2 backchannels per minute in, respectively, American English and German conversations and Heldner et al. (2013) reports backchannels corresponding to 8.8 (vocal) and 13.0 (vocal, visual or multimodal) backchannels per minute in Swedish. Mowlaei (2017), on the other hand, only found backchannels corresponding to an average frequency of 1.1 backchannels per minute in Persian conversations. The number of backchannels in French neurophychological tests found by Bailly et al. (2016) corresponds to about 4.9 backchannels per minute, illustrating the potential influence of task as well as language. For the study below, it is hence plausible that speakers with different first languages (L1) will have differing views on how frequently a robot should provide backchannels to be perceived as attentive.

#### 2.1.3 Timing of backchannels

Since the role of backchannels is to support the speaker in the conversation, they should be provided when they would be welcomed by the speaker, which is signalled by, e.g., pauses, a change of pitch (Ward et al., 2007), head movements or gaze. Heldner et al. (2013) proposed that conversations include backchannel relevance spaces, during which it would be appropriate for the listener to produce backchannels. Using an audiovisual corpus, they showed firstly that these spaces were much more frequent than the actual backchannels provided and secondly that head nods without verbal backchannels constituted 20%–53% of the backchannels. The latter underlines the potential benefits of using a robot compared to a voice assistant for natural conversations, since the robot can provide multi-modal and alternate-mode backchannels. Inden et al. (2013) further showed that verbal backchannels are more frequent towards the end of the speaker’s utterance, but that visual backchannels are provided more uniformly over the speaker’s utterance.

### 2.2 Backchannels in HRI

Research on backchannels by robots and virtual agents is primarily focused on backchannel opportunity prediction (BOP), i.e., frequency and timing of backchannels and, more seldom, backchannel category prediction (BCP), i.e., backchannel formulation. In addition, human provision of, reaction to and perception of backchannels in HRI have also been studied.

#### 2.2.1 Frequency and timing of backchannels

Park et al. (2017) showed that a toylike robot that provided backchannels based on identified backchannel opportunities offered by 4–6 year-old children telling stories had a positive impact on the children’s perception of the robot and that the children preferred telling stories to this robot compared to one that did not provide backchannels. In a perception experiment with an animated listener that provided backchannels differing in quantity, timing and form, Poppe et al. (2011) showed that both too frequent and too seldom backchannels had a negative impact on the perception of the agent and further that head nods were often seen as more appropriate than verbal backchannels. Similarly, Inden et al. (2013) assessed, using a virtual embodied conversational agent (ECA), if timing backchannels with the speaker’s pauses, utterance units and/or rhythmic pattern was perceived as better than randomly provided backchannels. Overall, these timing strategies were not as superior to random backchannels as were those copied from a human listener, except regarding fewer missed backchannel opportunities. To find appropriate backchannel timings, Hussain et al. (2019) proposed to use reinforcement learning of a Markov decision process, but they never actually tested the learned behaviour in HRI; Murray et al. (2022) did find that a data-driven approach using a long term short term memory recurrent neural network to learn head-nod backchannels for a robot listening to a human speaker was preferred over both random nodding and a rule-based backchannel model. The present study instead uses simpler, rule-based heuristic approach that distributes backchannels during and after speaker turns (Section 3).

#### 2.2.2 Formulation of backchannels

Different backchannel formulation methods have been implemented and tested in HRI, from copying the actual backchannels of a human listener in the same conversation (Gratch et al., 2007; Poppe et al., 2011; Blomsma et al., 2022), over learning from observing human listeners and formulating rules (Gratch et al., 2007; Al Moubayed et al., 2009) or from data using machine learning approaches (Adiba et al., 2021), to hand-crafted backchannels deemed suitable for the robot at hand (Park et al., 2017; Murray et al., 2022). Backchannels specifically created for robots include head nods (Fujie et al., 2004; Al Moubayed et al., 2009; Park et al., 2017; Murray et al., 2022), gaze and eyebrow movement (Park et al., 2017), smiling (Al Moubayed et al., 2009; Park et al., 2017) and short utterances (Fujie et al., 2004; Park et al., 2017; Sebo et al., 2020). Adiba et al. (2021) proposed an early loss function to train an attention-based long short-term memory model to predict the category of Japanese responsive or expressive backchannels, together with the opportunity to provide them. The present study uses a fixed set of verbal and visual backchannels, as described further in Section 3.

#### 2.2.3 Perception of robot backchannels

As we will investigate the interlocutors’ perception of the robot, it is relevant to consider the study by Gratch et al. (2007), which investigated the rapport between human subjects and a virtual agent that used backchannels. The virtual agent was compared with human-human face-to-face interaction in three conditions, with the agent’s response copied from a human, automatically created to respond to the interlocutor or non-aligned with the backchannel opportunities provided by the speaker. It was found that the rapport was as strong with the responsive agent as with a human listener and stronger than both with the copied and non-matching behaviour. If the robot’s backchannels are successfully provided, we can thus in general assume that subjects would have a positive perception of it. However, as the robot’s backchanneling strategy will not be tailored to subjects’ first language or other socio-cultural characteristics, what is a successful strategy with some users may be unsuccessful with others, which is why we aim at investigating the relationship between backchannel statistics, cultural factors and subject perception.

Al Moubayed et al. (2009) studied the same types of backchannels in the robotic dog Aibo and in an animated ECA and concluded that both the realisation of and reactions to the backchannels differed, compared to each other and compared to backchannels from a human listener, since the robot was physical but less human-like, while the ECA had a more human-like appearance and behaviour, but was virtual. In the experiment in this study, we are using the robot Furhat (Al Moubayed et al., 2012), which combines physical presence with substantially more human-likeness and facial expressiveness by using a back-projected computer-animated face (the robot is described further in Section 3.6). While no direct comparison is performed between responses to the robot’s backchannels and that of a human listener, it will nevertheless be of interest to consider if the expressiveness and human-likeness of the Furhat robot influence the responses to its backchannels, so that they are more similar to those in human-human interactions than what Al Moubayed et al. (2009) found.

Finally, as conversations are bidirectional exchange of information, it is also important how human interlocutors backchannel towards robots. Bliek et al. (2020) investigated how the Pepper robot’s backchanneling cues (pauses and gestures) influenced human listeners and how their backchannels differed compared to when directed towards a human interlocutor. It was shown that much fewer (3.7 times), and in particular fewer verbal, backchannels were provided to a robot speaker. In addition, the subjects rated the same task-based conversation with a human higher than with a robot.

### 2.3 Language aspects of backchannel generation and perception

Traditionally in research on backchannels, culture has been synonym with nationality and/or language, and most work has focused on differences between languages, related to how frequently backchannels are expected, how they are formulated and speaker cues signalling that a backchannel is welcome. Wong and Peters (2007) showed that there are differences in the distribution of backchannel use and complexity also between regional varieties of English (with Australian listeners providing more, but simpler, backchannels). We summarise findings on language influence on backchannels in terms of function in addition to frequency, timing and formulation.

#### 2.3.1 Functions of backchannels

The role of backchannels, and hence the distribution between different backchannel functions, clearly differs between different languages. Ferré and Renaudier (2017) reports that the distribution of vocal backchannels in English was continuer 31.2%, assessment 36.5%, agreement 11.6% and follow up 20.6%. This can be contrasted with the investigation of Wan (2018) of conversations between Hong Kong Chinese and Native English speakers. Based on the reported number of backchannels of different functions, frequencies can be calculated, and these show large differences between the groups of listeners: continuers (41.0% vs. 11.0%), understanding (27.3% vs. 18.2%), display of emotion or acknowledgement of newsworthy information (22.8% vs. 48.9%), change of activity (5.1% vs. 14.6%) and dispreference (3.7% vs. 14.3%). Another example is that Mowlaei (2017) found that the distribution of backchannels in Persian were display of understanding (22.5%), of agreement (12.9%), of emphatic support (9.6%), of emotion (16.1%), encouragement to continue (12.9%) or request for information and confirmation (25.7%). For Swedish, the target language in the present study, Fant (2015) found that display of attention and understanding dominate the verbal backchannels. What these studies show is hence that speakers of different L1s may have different expectations regarding what the backchannels should signal.

#### 2.3.2 Formulation of backchannels

Ward^1^ has compiled a list of frequently occurring backchannels in 21 languages as found in different studies. While some types of backchannels are frequent in many of the surveyed languages, in particular different forms of affirmation (*yes*, *si*, *oui* in English, Spanish, French) and non-lexical acknowledgement (*mm*, *uhu*), other types are found in much fewer languages, such as lexically negative backchannels to support a negative speaker utterance (*no*, *nee*, *nej* in, respectively, British English, Dutch and Swedish), backchannels that express surprise (*oh*, *really?*, *va?* in British English, Swedish) or laughter (Mexican Spanish, Japanese, Egyptian Arabic). It is clear that such differences can affect intercultural conversations, e.g., if the speaker interprets a lexically negative backchannel as disagreement or expressions of surprise as non-understanding. Moreover, the same forms may not convey the same functional meaning, as shown by Najim and Muhammad (2020) who found that Kurdish backchannels, even though similar to English in theory, were used differently, with some forms having multiple functions.

When it comes to the multimodal nature of backchannels, Kobayashi (2013) found large differences between the audiovisual nature of Japanese and English backchannels, with respectively, 22.5% *vs.* 40.3% of backchannels being non-vocal, 25% *vs.* 30.9% being vocal and 52.5% *vs.* 28.7% being combined vocal and non-vocal.

#### 2.3.3 Frequency of backchannels

As already described in Section 2.1, it is well-known that the frequency of backchannels differs between languages, and based on previous studies, Ward concludes that backchannels are very common in Japanese, fairly common in English, Dutch, Arabic, and Korean, less common in German and significantly less common in Chinese and Finnish. The high frequency of backchannels in Japanese has been corroborated by, e.g., White (1989) who found that the Japanese conversation structure and culture encourage more backchannels and that Japanese listeners produced more frequent backchannels than American in their respective L1 interactions. A similar difference was found between Japanese and British English listeners, who, respectively on average had 7.1 and 8.1 speaker words between their backchannels (Cutrone, 2005). Heinz (2003) found that German monolingual listeners produced significantly fewer backchannels than American English listeners and that these were overlapping less with the speaker’s turns.

Other studies, with different types of language families have shown that Pakeha listeners produce more backchannels than Maori listeners (Stubbe, 1998) and that the Ugandan tone languages Ruruuli/Lunyala have more than twice as many backchannels per minute as English (Zellers, 2021).

#### 2.3.4 Timing of backchannels

Heinz (2003) showed that German listeners’ backchannels overlap less with the speaker’s utterances than do American listeners’ and (Fant, 2015) similarly found that Swedish listeners mainly backchannel during pauses, leading him to conclude that Swedish can be described as a “turn-giving”, rather than a “turn-taking” language. However, even if English backchannels may overlap more with the speaker utterance, they are most often provided at the end of clauses or sentences, whereas Japanese listener backchannels to much less extent coincide with sentence-final pauses and instead align with the speaker’s head movements (Maynard, 1997).

#### 2.3.5 Backchannels in intercultural conversations

It has been emphasized that second language learners need to master backchanneling in the second language (Ward et al., 2007; Cutrone, 2010) and Li et al. (2010) showed that proficient Chinese speakers of English as second language (L2) did not transfer backchannel formulation from Mandarin to English. Cutrone (2019) similarly found that Japanese L2 English learners regarded to be more proficient in English had a lower frequency of backchannels and that these occurred more frequently at clause final boundaries of the speaker utterances. However, it was also found that all Japanese listeners had a low variation in backchannel formulation and that several of them actually used positive backchannels in situations of non-understanding. Purwanti (2018) showed that Indonesian L2 speakers of English primarily used backchannels to signal that they were attentive, supportive and polite towards Australian L1 speakers. They employed non-lexical backchannels such as *oh*, *ha*, *hmm yea*, *umm*, *yea aah*, *yeah*, *yeap*, *aha aha*, and lexical items such as *okay*, *right*, *all right exactly*, *okay*, *yes*, which are hence quite similar to the L1 speakers’ use of backchannels.

Since conversations are a two-way interaction, L1 speakers may also adapt backchannels when interacting with L2 speakers. White (1989) showed that American listeners adapted their backchannels to suit their L2 interlocutors better, while Japanese listeners maintained their backchannel strategy. It has further been shown that L2 backchannel conventions may influence the listener’s strategy in their L1. Heinz (2003) found that German listeners who were proficient in American English (in which backchannels are more frequent than in German) produced more backchannels in a German conversation than did monolingual Germans. Wolf (2008) further showed that the fluency of Japanese L2 speakers of English when they performed presentations increased when they were provided backchannels, hence demonstrating the important communicative role of backchannels in L2 conversations.

Related to the below study, which includes the human interlocutors’ perception of the robot, it is worth noting that White (1989) reported that unmatched backchannel strategies in intercultural American-Japanese did not lead to a negative perception of the interlocutor.

### 2.4 Socio-cultural aspects of backchannel generation and perception

There is evidence that other factors, such as gender and age, may also influence backchannels. Many studies (see Stubbe (2012) for a summary) have indicated that there are differences in how men and women use and interpret backchannels, with claims that women use more backchannels than men (Ueno, 2008), that they use laughter and smiles more and also that men and women interpret backchannels differently (Mulac et al., 1998). Early findings on differences between women’s and men’s backchannels have later been criticised on the basis that the studied conversations were on different topics, and that it was more natural and important to provide backchannels within the topics that the women in the studies talked about.

#### 2.4.1 Gender effects on backchannel frequency and formulation

Using an analysis of large radio dataset of 1800 listener backchannels in 22 conversations Stubbe (1998) showed that Pakeha and Maori men actually produced more backchannels than Pakeha and Maori women, but their backchannels were consistently more frequently neutral than supportive (60% *vs.* 40%), whereas the women’s were consistently more frequently supportive than neutral (65% *vs.* 35%). When it comes to perception of backchannels, Mulac et al. (1998) demonstrated that men perceive backchannels as more controlling (steering the conversation) or as signals of uncertainty than female, who instead to a larger extent interpreted them as a display of interest in the speaker’s information.

#### 2.4.2 Age effects

Kraaz and Bernaisch (2022) investigated backchannels by younger ( < 26 years) and older (≥26 years) speakers of British, Indian and Sri Lankan English and found that the younger listeners in all groups provided substantially more backchannels than the older. Smaller differences between genders were also observed, in that Sri Lankan and Indian younger males produced more backchannels than female listeners, while the opposite was true for all three groups of older listeners and, to some extent, for the younger British English listeners. Yamashita (2017) investigated backchannels in English and Japanese parent-children conversations and found that the frequencies of backchannels were similar for the two settings, with the adults providing 5 (Japanese) to 14 (English) times more backchannels than the children. The distribution of forms of backchannels differed, with English parents using almost three times more phrasal backchannels than the Japanese, while the latter instead used many more repetitions. Both groups used non-lexical backchannels most frequently, but the Japanese parents used almost as many repetitions as non-lexical backchannels. Bodur et al. (2022) also found socio-cultural effects related to age and interlocutor-familiarity in the rate and type (generic or specific) of backchannels in on-line conversations, as adult speakers used more backchannels of both types in conversations with adult non-family members (about 3.2 backchannels of each type per minute) than with adult family members (about 1.6 generic and 2.0 specific backchannels per minute) or with a child (about 1.2 generic and 1.8 specific backchannels per minute) and children favoured specific backchannels over generic (about 0.9 generic and 2.5 specific backchannels per minute). The substantially higher rate of backchannels—and also backchannel opportunities offered by the speaker—in conversations with a less familiar interlocutor is important for the present study as it may indicate a higher need to request and display listener understanding and attentiveness in interactions with unfamiliar interlocutors. Since robots are even more unfamiliar to many human speakers, a higher frequency of backchannels may be required for speakers who have little experience of robots.

## 3 Robot backchannels experiment

The experiment setup and focus follows closely on the one presented by Gillet et al. (2021), which investigated how gaze from a Furhat robot can balance participation of two human speakers with different linguistic proficiency in a triad word game. The motivation of the line of research is to provide conversation practice for L2 learners of Swedish using the game Taboo (see Section 3.1.1). The previous study mixed speakers of different linguistic proficiency by pairing L1 and L2 speakers and found that a Furhat robot could influence the less active speaker to speak more by increasing the visual attention towards this speaker. The present study builds upon an extension of that experiment (Cumbal et al., 2022), which investigated the influence of audiovisual backchannels instead of gaze only, and we here focus specifically on different socio-cultural characteristics of the speakers.

Based on the findings in previous work, described in Section 2, we thus investigate the main research question *Do socio*-*cultural factors*—*specifically gender*, *age*, *extroversion level and familiarity with robots*—*influence how L1 and L2 speakers respond to robot backchannels aimed at balancing the activity in multiparty interaction?*

The question is analysed in an experiment where a social robot provides relatively more, and more expressive, backchannels towards the least speaking participant in an interaction pair. Our hypotheses are that **H1**. The adpative robot backchanneling behaviour will, in general, make the participation more balanced than if the same backchannel strategy is used towards both speakers; **H2**. Socio-cultural characteristics of the speakers will influence, quantitatively, in the form of speaking time, and qualitatively, in the form of interaction responses, how effective the adaptive robot backchannel strategy is; and **H3**. Socio-cultural characteristics of the speakers will also influence how they perceive the robot’s interaction.

### 3.1 Experiment protocol and setup

The user experiment was conducted by two researchers, one with the main responsibility of interacting with the participants and one in charge of launching and monitoring the hardware and software for the experiment.

**FIGURE 1:**
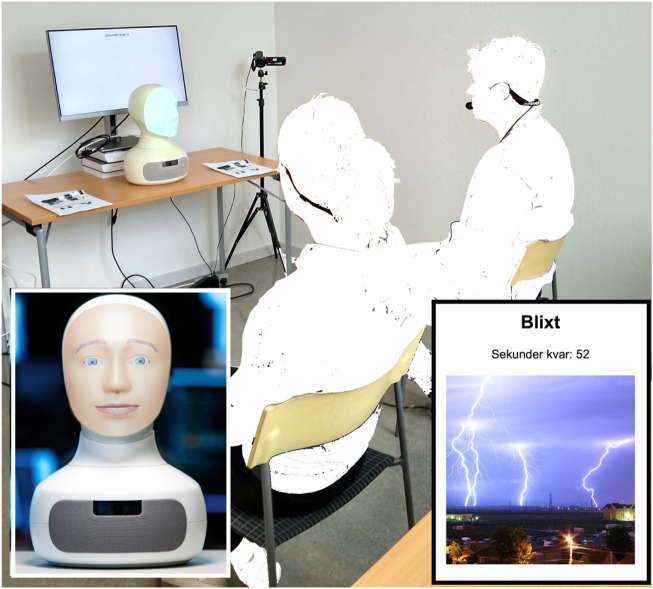
Experiment set-up showing the placement of the robot, the word game screen, anonymized participants and the front camera. Inlay, left: The Furhat robot. Inlay, right: Game card showing word, picture and number of seconds left.

The first researcher welcomed the participants, distributed and collected the informed consent form—which described the experiment, the data collection, the handling and analysis of the recorded data and the subject’s rights to withdraw from the study during or after the experiment—and checked that the participants had filled in the pre-study questionnaire (Section 3.3), which was distributed together with the confirmation of study participation. The participants were then taken to the lab and invited to take a seat in the respective chairs facing the robot Furhat with the *Titan* face mask, as shown in Figure 1. When the participants had taken on the head-mounted microphones, the experiment leader started the video camera capturing the scene from Furhat’s perspective, informed the participants that they were being recorded and that the robot would initiate the game, and then left the room.

The robot introduced itself, asked the participants some icebreaker questions and then repeated the rules of the game (see Section 3.1.1). This was followed by a short (less than 2 min) practice round, which was scripted and controlled by a wizard-of-Oz. In the practice round, the robot explicitly asked each participant to describe a word shown on screen until the robot had guessed the correct word. The game interaction, in which the robot was autonomous, then lasted about 15 min. After the robot had terminated the game interaction with the two participants, the participants were taken to separate locations to fill in the post-session questionnaire (Section 3.4) and were thanked for their participation with a gift voucher corresponding to approximately 10$.

#### 3.1.1 Task description

The human-robot interaction was centered around the word guessing game Taboo, in that the human players should describe a word—without saying the word or part of it—shown to them, in writing and with a picture, on a computer screen. The screen was placed behind the robot, as shown in Figure 1, and the picture was hidden after 15 s, to encourage the subjects to focus on the robot rather than on the game screen. The implementation^2^, done in the Robot Operating System (ROS) framework, is described in more detail by Gillet et al. (2021) and Cumbal et al. (2022), but in summary it is based on the commercial Swedish version of the game, “Med Andra Ord”, from which five target words each on the easy and medium levels and at least one on the hard level were selected. Each game round, corresponding to one word to be described, lasted until the robot “guessed” the correct word (see Section 3.6) or for a maximum of 60 s. The number of game words (11+) and the maximum time (60 s) per game word limited the total interaction time. The participants were informed that the robot listened to both participants all the time, without any separate player turns and they were not instructed regarding if they should collaborate or compete in the game. Consequently, no points or rewards were given for successful robot guesses.

### 3.2 Backchannel manipulation

Two sets of robot backchannel behaviour were used in this experiment, the rule-based static (for the control group) and the activity-adapted (for the experimental group). The two sets are equal in terms of backchannel verbal formulation and timing, and to the extent that it is possible to control, target for total frequency of feedback during the session (note that the amount of backchannels depends on the subjects’ speaking activity and therefore differs between sessions). The two sets are therefore regarded to be equally valid robot backchanneling strategies in general (as opposed to if the baseline was, e.g., no or randomly distributed backchannels, which has often been used as baseline in previous studies). The difference between the two sets primarily lies in how they are distributed between the two human interlocutors.

#### 3.2.1 Control group robot backchannels

The robot used the “interested” and “understanding” backchannels in the Acapela text-to-speech synthesis for Swedish with the Elin voice. A large variation of predefined non-verbal (e.g., “aa”, “å”, “mm”) and verbal (e.g., corresponding to “yes”, “yeah, go on”, “no”) in different prosodic renditions are available. For the control group backchannels, a low intonation and a neutral emotion expression was used and no additional visual backchannel cues were provided.

Backchannels were provided at approximately 35% of all backchannel opportunities, which where defined heuristically during pilot testing so that they occurred during the human speaker utterances with a minimum interval of 2.9 s between backchannels and only for utterances longer than 1.5 s, but without any linguistic analysis of the semantics of the speaker’s utterances. Since the robot automatically turns its head towards the active speaker, backchannels are clearly addressed towards that speaker.

This control condition hence corresponds to a backchanneling behaviour that has been found to be preferred over completely random or no backchannels in previous HRI studies. The average number of backchannels per speaker in the Control condition was M = 11.7 (SD = 13.6), corresponding to 3.7 backchannels per minute of active speaking time.

#### 3.2.2 Experimental group robot backchannels

The adaptive robot backchanneling for the experiment group used the same formulation and identification of backchanneling opportunities as for the control group, but provided backchannels inversely proportional to the interlocutor’s accumulated relative speaking time. That is, the interlocutor that has spoken the least should receive more frequent and more expressive backchannels, with the aim of increasing the speaking share for that interlocutor by displaying a boosted listener interest by the robot. Since the accumulated speaking time is used to determine the more and less active interlocutors, the robot’s backchannel strategy is initially attuned and its decision regarding which interlocutor should receive more backchannels may hence change during the interaction. On average, the least active speakers received 2.1 as many backchannels per speaking time in the Experimental condition compared to the Control, whereas the more active speakers received fewer (0.85 times) backchannels per speaking time in the Experimental condition. The encouraging backchannels were selected to be more expressive and energetic and were accompanied by a smile or an affirmative nod.

The average number of backchannels per speaker in the Experimental condition was M = 13.3 (SD = 11.6), corresponding to 5.3 backchannels per minute of active speaking time.

### 3.3 Pre-session questionnaire

The pre-session questionnaire gathered demographic information (age, gender, country of origin, first language, proficiency in Swedish, age of starting to learn Swedish, other languages spoken at a high proficiency level and educational level), determined the subject’s extroversion level using eight questions from the Big Five Inventory scale (John et al., 1991) and chartered the experience and acceptance of robots using nine questions.

The questions about robots were; regarding experience: “*I am using the following voice assistants on a regular basis*” [*Apple Siri*, *Google Assistant*, *Google Home*, *Amazon Alexa*, *Other*, *None*], “*Describe your previous experience of interacting with a speaking robot*”; and attitudes: “*Describe your general attitude towards interacting with a speaking robot*”; “*In my opinion*, *robots should be developed to be as human*-*like as possible when it comes to* … *appearance*/…*interaction*/…*voice and spoken utterances*/…*understanding speech*,/…*emotions*” and “*I think that it would be a good idea to employ robots* (*now or in the future*) *in* (*choose all you agree with*)” [*Education, Entertainment* (*e.g., social interaction*)*, Service sector* (*e.g., providing information*), *Domestic* (*e.g., cleaning*)*, Healthcare* (*e.g., social companion or screening of patients*)*, Medicine* (*e.g., surgery*)*, Industry, Military/Security, None of the above*]. Unless other answer alternative are stated above, all questions were to be answered on a five-point Likert-scale 1–5 with guiding descriptions for each level and 1 being very low, 3 neutral and 5 very high.

### 3.4 Post-session questionnaire

The post-session questions focused on the subject’s impression of the robot in the interaction: “*I felt that the robot understood what I was saying*”, “*The robot was paying attention when I was speaking*”, “*The robot was friendly towards me*”, “*The robot was introvert/extrovert*”, “*The robot was balancing its attention between me and my partner*” and “*The robot behaved like a human listener*”. All questions were answered on a five-point Likert scale 1–5 with guiding descriptions for each level, with 1 and 5 respectively signifying very low and very high levels and 3 neutral.

### 3.5 Subjects

Participants were recruited on the campuses of KTH and Stockholm University, using flyers and electronic invitations. In total, 38 adult subjects, 15 men, 22 women and 1 not disclosed were recruited, with the demographic details indicated in Table 1. 20 subjects were L1 speakers of Swedish, whereas the L1s for remaining 18 subjects were English (3), German (2), Dutch (2) [Germanic languages]; French, Italian, Romanian [Roman languages]; Bulgarian, Polish, Russian [Slavic]; Greek; Farsi (2) and Chinese (3). The self-reported proficiency levels in Swedish of the L2 speakers were 7 advanced, 10 intermediate and 1 elementary.

**TABLE 1:** Summary of demographic details and pre-session answers. L1 groups are explained in the main text. M,F indicate the number of Male and Female subjects (* = +1 non-binary). Extroversion, Attitude towards interacting with robots and ratings of how human-like the robot should be when it comes to Appearance, Interaction, Speech, Natural Language Understanding (NLU) and Emotion display are means and standard deviation on a five point Likert scale 1–5.

		Age	Extro-	Attitude		The robot should be human-like regarding			
L1	M,F	(yrs)	vert	to robots	Appearance	Interaction	Speech	NLU	Emotion
Swedish	8,11	34 ± 13	3.1 ± 0.8	3.4 ± 0.7	2.6 ± 1.0	3.5 ± 0.8	3.7 ± 0.9	4.4 ± 0.9	2.5 ± 0.7
Germanic	3,3*	34 ± 12	3.6 ± 0.9	3.9 ± 0.9	2.9 ± 0.4	3.4 ± 1.0	3.9 ± 0.9	4.6 ± 0.5	3.1 ± 0.9
Roman	0,3	35 ± 12	3.4 ± 0.5	3.0 ± 1.0	3.0 ± 1.0	4.3 ± 0.6	4.7 ± 0.6	5.0 ± 0.0	3.3 ± 0.6
Greek	0,1	34	2.8	3.0	2.0	4.0	2.0	4.0	2.0
Slavic	2,1	35 ± 5	2.8 ± 0.2	3.7 ± 1.5	2.0 ± 0.0	4.3 ± 1.2	3.3 ± 0.6	4.7 ± 0.6	2.3 ± 0.6
Farsi	1,1	40 ± 3	4.0 ± 0	3.0 ± 0.0	3.0 ± 0.0	3.0 ± 0.0	3.5 ± 0.7	4.0 ± 1.4	3.0 ± 0.0
Chinese	1,2	29 ± 5	2.5 ± 0.7	3.3 ± 0.6	3.0 ± 1.0	3.3 ± 1.2	3.3 ± 0.6	4.3 ± 0.6	2.7 ± 1.2
	15,22*	34 ± 11	3.2 ± 0.8	3.4 ± 0.8	2.7 ± 0.8	3.6 ± 0.9	3.7 ± 0.9	4.4 ± 0.8	2.7 ± 0.8

Most subjects had no (13) or little (11) experience of interacting with robots, while 12 had some experience and 2 were regular users (M = 2.1,SD = 0.9 on a scale from 1 = “None” to 5 = “Expert”). The L2 speakers were slightly more familiar with robots (M = 2.2,SD = 0.9) than the L1 speakers (M = 1.9,SD = 1.0). Further, most (24) did not use any voice assistant, and of those who did, Apple’s Siri was the most used (4 subjects), followed by Google Assistant (3), Google Home (3) and Amazon Alexa (2). The subjects were rather neutral regarding general attitude towards interacting with robots (M = 3.4, on a scale from 1 = “Very uncomfortable” to 5 = “Very relaxed”).

With two exceptions, no significant differences were found, using non-parametric Mann-Whitney U-tests, in the pre-session responses between L1 and L2 speakers, between men and women, between “younger” (below the average of 34 years) and “older” (above or equal to 34 years) or between subjects who had less (below or equal to the average of 2.0) and more (above 2.0) experience of robots. The only significant differences (*p* = 0.02) related to the previous experience of interacting with robots, with the younger and male participants having more experience (*M*_*young*_ = 2.4,SD = 0.89 and *M*_*male*_ = 2.6,SD = 0.94) than, respectively, the older and the female (*M*_*old*_ = 1.7,SD = 0.82 and *M*_*female*_ = 1.8,SD = 0.80).

As shown in Table 1, the subjects agreed the most with the statement that the robot’s natural language understanding should be human-like, followed by natural spoken utterance generation and interaction, whereas the overall preference was that the robot’s appearance and emotion display should not be human-like (scale from 1 = “Strongly disagree” to 5 = “Strongly agree’). Since this study focuses on signalling robot understanding and doing so in an intuitive manner for the interaction, the pre-session answers indicate that it is of importance that the robot’s backchannels are perceived as human-like. Using robots for education (i.e., the area of application in this study), was considered to be good by 68% of the respondents, which was the fourth highest ratio, after industry, domestic and service sector (all 87%).

The participants were paired so that one L2 Swedish speaker played the game together with either an L1 speaker of Swedish (15 interactions) or another L2 speaker (one interaction with two C1 level subjects and one B2-C1 interaction). Three sessions, two in the Experimental condition and one in the Control, were with two L1 speakers. The pairing was balanced as much as possible between the two experiment groups when it comes to gender (Control: 12 female and 6 male, Experimental: 11 female, 9 male, 1 N/A), proficiency (Control: 8 L1 speakers, 5 advanced, 5 intermediate; Experimental: 12 L1 speakers, 2 advanced, 5 intermediate, 1 elementary) and age (Control: *M*_*C*_ = 34.5 years, SD = 13.1; Experimental: *M*_*E*_ = 32.4 years, SD = 9.5). Extroversion (*M*_*C*_ = 3.38, SD = 0.79; *M*_*E*_ = 3.09, SD = 0.61), familiarity with robots (C*M*_*C*_ = 2.1, SD = 1.0; *M*_*E*_ = 2.1, SD = 0.85) and attitudes towards robots (*M*_*C*_ = 3.63, SD = 0.88; *M*_*E*_ = 3.31, SD = 0.87) were not controlled for, since the pre-session questionnaire data was analysed after the experiment, but were nevertheless rather balanced between the two conditions.

### 3.6 Game interaction with the robot

This study takes advantage of several of the rather unique features of the robot used, Furhat from Furhat Robotics, in order to be able to create realistic backchannels and a natural spoken interaction. Firstly, Furhat combines the expressiveness and flexibility of virtual conversational agents and the physical presence of robots, by back-projecting a computer-animated face on an interchangeable 3D mask (Al Moubayed et al., 2012) fitted on a neck with three moto-servos allowing the robot to turn, roll and tilt its head. For our study, this is utilized to display positive facial expressions (e.g., smiles, eye and eye brow movements) and head nods, which are essential components in human backchanneling, as shown in Section 2.1. The robot further automatically turns its head in the direction of the speaking player, based on direction-sensitive speech activity detection, with a restriction of a minimum of 2 s gaze fixation to avoid too frequent shifts. Such head turning behaviour has been shown to create successful multiparty HRI (Skantze et al., 2015). Directional microphones and a wide-view camera incorporated into the robot’s bust allow Furhat to determine the active speaker and meet that person’s gaze based on face tracking through computer vision.

Secondly, the modular text-to-speech synthesis component, which here used a female voice from Acapela, is automatically supplemented by accompanying realistic lip movements, which are an important support for L2 learners’ speech perception. Furhat software further supports natural language understanding using real-time cloud-based automatic speech recognition for its interaction with users. However, due to generally poor automatic speech recognition performance for L2 speakers of Swedish (Cumbal et al., 2021), its guesses were not based on a linguistic analysis of the players’ hints, but instead semi-randomly selected from a pre-generated list of plausible alternatives for each game word. The correct word was included in the list and the likelihood of selecting the correct word increased with time within each game round of 60 s. This hence means that the robot’s guesses were always sensible in general, but were not directly linked to the hints, which may make the robot seem less knowledgeable (e.g., guessing *mustard* or *Hollandaise sauce* when the hint was that it is a sauce made of tomatoes), but this was still appropriate for the purpose of the interaction (in fact, it is even an advantage that the subjects need to try different hints and that robot does not end the game round too quickly by guessing correctly).

The game interaction, in which the robot was fully autonomous, may be illustrated through the examples in Tables 2, 3. These examples of the interlocutors’ descriptions and the robot’s guesses and backchannels are discussed in more detail from an interaction perspective in Section 4.2. While the robot could provide backchannels during a player’s utterance (see line 9–10 in Table 2), it never interrupted with guesses, but waited for the player to complete its turn.

**TABLE 2:** Transcript excerpt from a high collaboration interaction. Sp = Speaker (Left, Right, Furhat). Robot backchannels highlighted in bold.

#	Sp	Utterance	Embodied Action	English Translation
01	L:	en sten (0.6) som används (0.8) typ mycke	L and R mutual gaze	*a stone which is used like much*
02		(0.8)		
03	L:	till exempel,		*for example*
04		(1.0)		
05	L:	eller? va använder man de till?	L turns to R	*or? what do we use it for?*
06	R:	hahahaha ja vet inte	R looks at L; mutual gaze; F looks at R	*hahahaha i do not know*
07	L:	hihihi	L and R laugh together	
08	R:	erm: jätte::		*much::*
09	L:	ädelst [en eller]?		*gemstone or?*
10	F:	**[uhum]**	F turns to L	
11	L:	ä den ädelsten?		*is it gemstone?*
12	R:	a:: ja tror så		*yeah:: I think so*
13	F:	**uhum**	F turns to R	
14	R:	erm:		
15	L:	den ä brun?		*it is brown?*
16	R:	°den ä brun°		*it is brown*

**TABLE 3:** Transcript excerpt from a non-collaborative interaction. Sp = Speaker (Left, Right, Furhat). Robot backchannels highlighted in bold.

#	Sp	Utterance	Embodied Action	English Translation
01	R:	e: en sport	R and F mutual gaze	*e: a sport*
02	F:	**uhum.**		
03	R:	som: (0.2) e brukar (0.3) associeras me friidrott.		*tha:t e is used to be associated with athletics*.
04	F:	**uhum.**		
05		(4.0)		
06	F:	e:: maraton,		*e:: marathon*,
07		(5.0)	L looks at F; R gazes shortly toward L	
08	L:	.HH	a deep inbreath; moves his feet forward	
09		(2.0)		
10	L:	tsk	smacks his lips	
11		(4.0)	L cracks the knuckles; R looks at L; F turns to L	
12	R:	e::	F turns to R	
13		(3.5)	R drums with her fingers on her knees	
14	L:	en svensk hade länge rekordet.	F turns to L	*a swede long had the record*.
15	F:	**uhum**	F nods	
16	L:	I denna gren.		*in this branch*.
17	F:	a: stafett?		*a: relay?*

### 3.7 Measurements and data analysis

The measurements consisted of interaction data and the participant responses to the post-session questionnaire. The interaction data was firstly each participant’s accumulated speaking time, as detected by the speech activity detection system and secondly video recordings of the scene. The subjects’ speech and speaking time were recorded with individual head-mounted *Shure Model WH20* professional microphones that were calibrated to avoid detecting non-speech content like sudden movements, breathing, or general noise. The accumulated speaking time was measured continuously during the experiment in order to adjust the robot’s backchannels towards the least active speaker in the Experimental condition. For the post-session analysis, the speaking times were used to investigate the influence the robot’s backchannel strategy had on the balance of the interaction (Section 4.1) and the video recordings were used for an ethnomethodology and multimodal conversation analysis (EMCA) (Mondada, 2019) (Section 4.2).

Three measures of speaking activity were used: the subject’s total speaking time *T*_*s*_ during the game, the ratio *R*_*s*_ of the subject’s speaking time divided by the total game time *T*_*tot*_ (*R*_*s*_ = *T*_*s*_/*T*_*tot*_, where *T*_*tot*_ includes the robot’s speaking time and pauses as well as the speaking time of the two subjects) and the distribution ratio *r*_*s*_ of the two subjects’ share of the speaking time (i.e., $r_{s}=T_{s}/\sum_{i=1}^{2}T_{s\left(i\right)}$ ). *T*_*s*_ measures the subject’s absolute speaking activity and *R*_*s*_ and *r*_*s*_ the relative activity, compared respectively to the entire session and to the other subject. Both *R*_*s*_ and *r*_*s*_ are used, since *R*_*s*_ is a more suitable measure of actual activity during the game (a game with two equally inactive subjects would result in a low *R*_*s*_ but an *r*_*s*_ close to 0.5), while *r*_*s*_ is a more direct measure of the balance between the two subjects.

In addition, the post-session questionnaire was used to identify how socio-cultural factors, identified through the pre-session questionnaire, influenced the participants’ reactions to robot backchannels (Section 4.3). For the analyses of differences in post-session survey responses, non-parametric Mann–Whitney U-tests were used to test for significance.

## 4 Results

From the pre-study, the following socio-cultural characteristics were identified as potentially influencing the responses to and perception of robot backchannels: first language (L1 *vs.* L2 speakers in this study), gender, age, extroversion and familiarity with robots. We investigated the influence of these socio-cultural factors on the balancing effect of the robot’s adjusted backchannel strategy (through differences in speaking ratios, Section 4.1), on how individual subjects responded to robot backchannels (through conversation analysis, Section 4.2) and on how they perceived them (through the post-session survey, Section 4.3).

### 4.1 Balancing uneven participation with different socio-cultural groups

**FIGURE 2:**
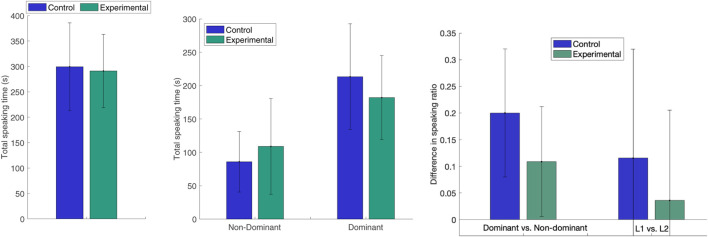
Left: Average total speaking time for both speakers. Middle: Average total speaking time for the dominant and non-dominant speaker in the pair. Right: Average difference in speaking ratio Δ*R* between the dominant and non-dominant speaker or the L1 and L2 speaker in each pair.

The aspect of how the robot’s vocal and visual backchannels influence the balance in the speakers’ participation is the specific focus of the study by Cumbal et al. (2022) and further details are available in that publication. We here replicate the main analysis for all 19 conversations, as Cumbal et al. (2022) only studied the 15 L1-L2 speaker interactions. We find the results summarised in Figure 2. Firstly, that the joint total speaking time for both speakers in a pair (*T*_*s*1_ + *T*_*s*2_) was very similar between the Experimental condition and the Control (which is to be expected with the current game setup that limits the total speaking time, since there is a maximum game time per word and a semi-fixed number of game words). Secondly, that the mean speaking time of the less active speaker in each pair was 26.8% higher in the Experimental condition than in the Control (*M*_*E*_ = 109.0, SD = 71.8 *vs.* *M*_*C*_ = 86.0, SD = 45.4). Thirdly, that the speaking time of the more active speaker was 14.8% lower in the Experimental condition than in the Control (*M*_*E*_ = 182.1.0, SD = 63.0 *vs.* *M*_*C*_ = 213.5, SD = 79.3).

The difference in speaking time ratio Δ*R* between the dominant and the non-dominant participant in the pair in the Experimental condition was almost half of that in the Control (mean difference *M*_*E*Δ*R*_ = 0.11, SD = 0.10 *vs.* *M*_*C*Δ*R*_ = 0.20, SD = 0.12), but this difference was not significant (Mann-Whitney U-test, *p* = 0.13). We ensured that the difference between the two conditions is not due to lower speaking ratio differences for the L1–L1 interactions by calculating the mean difference in speech ratio when excluding the three L1–L1 interactions (*M*_*E*Δ*R*_ = 0.12, SD = 0.10; *M*_*C*Δ*R*_ = 0.20, SD = 0.13). Overall, there was almost no difference in speaking ratio *R*_*s*_ between L1 and L2 speakers for the subset of 15 L1–L2 interactions in the Experimental condition (*M*_*E*Δ*R*_ = 0.03, SD = 0.17; *M*_*C*Δ*R*_ = 0.12, SD = 0.20). The reason for this difference being lower than that for dominant *vs.* non-dominant speakers, is that the L2 speaker was more active than the L1 speaker in three interactions in the Experimental condition and one in the Control.

**FIGURE 3:**
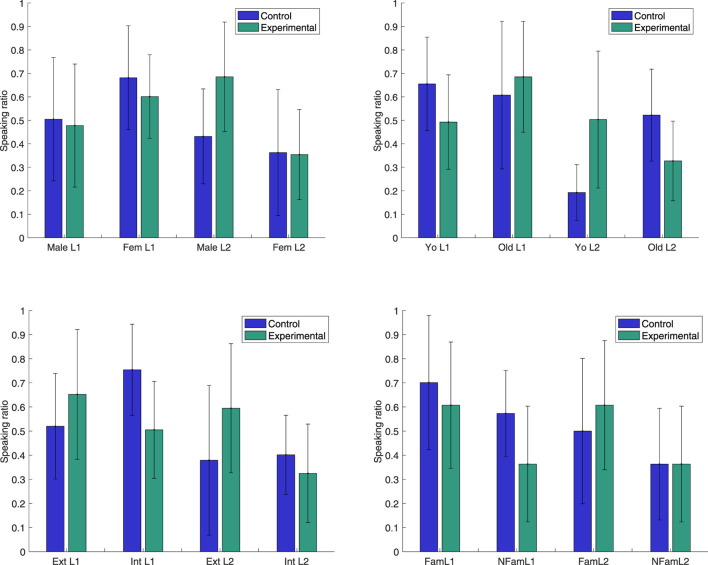
Speaking ratios *r* in Control and Experimental conditions depending on L1 or L2 speaker, in combination with Upper left: male or female (Fem), Upper right: younger (Yo) or older (Old) than the mean age in the group, Lower left: more (Ext) or less (Int) extrovert than the mean level in the group or Lower right: more (Fam) or less (NFam) familiar with robots than the mean level in the group.

These results demonstrate that the robot’s adaptive backchannel strategy reshaped the interaction, as it encouraged the non-dominant speaker to talk more and the dominant to talk less. However, hypothesis H1 was not formally confirmed, as the difference in speaker ratio imbalance was not significant. A main reason for this, illustrated by the high standard deviation in speaking time for the less active speaker in the Experimental condition, is that the encouraging effect was very different for different speakers. For this study, we therefore explore the data further by considering the differences in speaking time ratio *r*_*s*_ depending on gender, age, extroversion and familiarity with robots in combination with if the subjects are L1 or L2 speakers, as shown in Figure 3. L1 *vs.* L2 is considered as the main factor, since it, in addition to being a cultural feature in itself, corresponds to a difference in linguistic proficiency for 15 out of 19 interactions. We hypothesise that this difference leads to unbalanced speaking time, and hence difference in robot backchannel strategy in the experimental condition. As the subjects are then divided into eight sub-groups, they are too few per group to expect that statistical significance could be found (*p*-values for Mann-Whitney U-tests, all non-significant, are reported below). Since this is a between-subject experiment, comparisons between the Experimental and Control conditions are moreover made on the group level, and do hence not show actual changes in response for individual subjects.

Nevertheless, the observed mean decrease in speaking ratio difference Δ*r* between the non-dominant and dominant speaker in the pair points to possible relationships that are interesting to investigate further, both qualitatively (Section 4.2) and quantitatively (in future work with larger or more homogeneous subject groups):The *Male* L2 subjects in the Experimental condition are substantially more active than in the Control (*p* = 0.23), whereas female L2 speakers (as well as the L1 speakers) have a very similar speaking ratio in the two conditions (*p* = 0.93).The *Younger* L2 subjects in the Experimental condition are substantially more active than in the control (*p* = 0.11) and the opposite is to some extent true for younger L1 subjects (*p* = 0.17). Older L2 subjects have a lower speaking ratio in the Experimental condition than in the control (*p* = 0.26) and the opposite is true for older L1 subjects (*p* = 0.40).The more *Extrovert* L2 subjects in the Experimental condition are substantially more active than in the Control (*p* = 0.29), whereas the more introvert L2 subjects are slightly less active (*p* = 0.69).The more *Introvert* L1 subjects in the Experimental condition have a lower speaking ratio than in the Control (*p* = 0.10), whereas the group of extrovert L1 speakers have a slightly higher speaking ratio in the Experimental condition than in the Control (*p* = 0.63).*Familiarity* with robots may influence the reaction to adaptive robot backchannels, as L1 and L2 speakers who are more familiar with robots have a higher speaking ratio in the Experimental condition than those who are less familiar (*p* = 0.17). The L1 speakers who are less familiar with robots have a lower speaking ratio in the Experimental condition than in the Control (*p* = 0.17), whereas L2 speakers have a slightly higher speaking ratio (*p* = 0.80) if they are more familiar with robots, but no difference if they are less familiar with robots (*p* = 1.0).

What we observe would hence suggest that of the L2 speakers, the male, the younger, the more extrovert and the ones more familiar with robots are more encouraged by the additional backchannel attention from the robot, while the female, older and more introvert L2 speakers are not as encouraged to speak more, or may even speak less. For the L1 speakers, the younger, the more introvert and the ones less familiar with robots may be more susceptible to speaking less if the robot focuses its backchannels more towards the other interlocutor. Without a larger subject group, for which statistical relationships might appear, these observations are rather speculative, but it does seem plausible that extrovert subjects become more active when receiving more attention and that introvert subjects become less active when receiving less. Familiarity with robots could influence in terms of speakers who are more familiar with robots firstly are speaking relatively more in general (FamL1 and FamL2 in Figure 3), since they are more at ease, and secondly responding more to additional encouragements from the robot (FamL2), whereas less experienced speakers may speak less in general (NFamL1 and NFamL2) and not respond to additional backchannels (NFamL2) or diminish their speaking time more (NFamL1), if they hesitate to actively take the turn when the robot is focusing on the other interlocutor. It should further be noted that familiarity with robots is correlated with age and gender, as already mentioned in Section 3.5, and these categories hence overlap.

Multiple linear regression analyses were performed to investigate how the socio-cultural factors influenced speaking time. For the L2 speakers, there was a statistically significant (*p*-value = 0.0053) positive linear relationship between age and speaking time in the Control (T = −170.0 + 8.5*age, *R* ^2^ = 0.6), i.e., older L2 speakers spoke more. A linear relationship was also identified for extroversion level (the more extrovert subjects had a tendency to speak more, regardless of condition), but the effect size was too weak (*R* ^2^ ≈ 0.1) to be considered.

The above results are thus in line with H2, but few significant differences were found, which is unsurprising due to the low number of subjects per category. To explore H2 further and to analyse how different individuals in general responded to the robot’s backchannels, we perform a conversation analysis, as outlined in the next section.

### 4.2 Culturally-linked interaction responses to robot backchannels

The EMCA analysis was performed in two steps. First, high level annotation was made of if the speaker was holding or yielding the turn for different backchannel timings for 15 conversations (9 in Experimental and 6 in Control condition), excluding the conversations in which one or both participants spoke so little that no backchannels were generated.

A three-level backchannel timing annotation scheme was used: *Mid-turn*, (*close-to-*) *Turn-final* and *After-turn*. To exemplify these timings, in the utterance describing *lightning*: *“It thunders in the dark* **(MT)** *and then it suddenly* **(TF)** *becomes light.* **(AT)**”, (MT) is Mid-turn, (TF) is Turn-final and (AT) is After-turn. The latter could be with or without a preceding pause. The robot vocal backchannels were annotated as being of three different types, “*uhum*”, “*mm*” and “*aa*”, but as “*uhum*” dominated heavily (153 occurrences in Control and 357 in Experimental condition, compared to 38 “*mm*” in each condition and “*a*” only occurring 18 times in Experimental condition), differences in vocal backchannel formulation were not considered.

**FIGURE 4:**
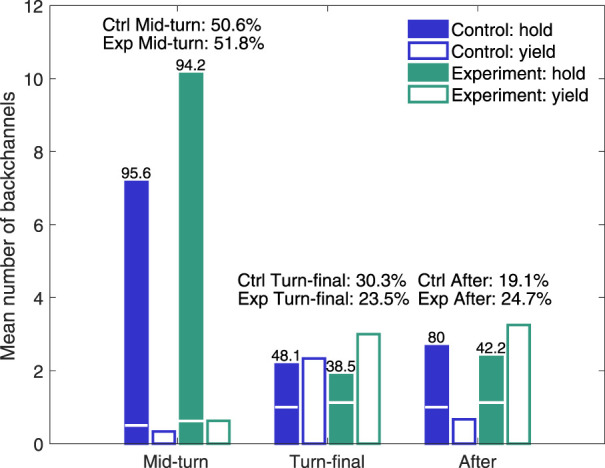
Mean number and percentage of speaker holding and yielding turn depending on backchannel timing. Numbers on top of Hold bars are the percentage of Hold vs. Yield for the different timings. The horizontal white line in the Hold bars indicate the mean number of speaker utterances that were preceded by a hesitation or pause (below line). The distribution between the different backchannel timings is given above the corresponding bar groups.

For speaker reactions, four different alternatives (two each for hold and yield) were annotated: Hold, when the speaker continued without or after a hesitation/pause and Yield, when either the other speaker or Furhat took the turn. This high-level annotation is summarised in Figure 4, which shows that, in addition to the already described increase in the number of backchannels in the Experimental condition compared to the Control, there is a clear difference in hold-yield pattern for the Turn-final (38.5% Hold in the Experimental condition *vs.* 48.1% in Control) and After-turn backchannels (42.2% Hold *vs.* 80.0%). The turn is hence yielded almost twice as frequently in the Experimental condition than in the Control for After-turn backchannels and 25% more frequently for Turn-final backchannels.

The total bar heights in Figure 4 display Hold numbers without considering if the backchannel or the speaker turn was preceded by a pause. For the Final-turn and After-turn backchannels in the Experimental condition, 60.0% and 47.3% of the speaker hold utterances were preceded by a pause, compared to 6.2% of the Mid-turn backchannels. The effect of a pause preceding the backchannel was marginal regarding if the speaker then hesitated or not before taking the turn (50% with pause *vs.* 44% without for the After-turn backchannels).

The result that speakers more often yield the turn than holding it after an After-turn backchannel may seem quite non-intuitive, since backchannels in one-to-one interactions usually encourage the active speaker to continue, but it seems that, in this three-party (game) setting, the Turn-final and After-turn backchannels may instead be perceived as an acknowledgement that the robot has understood the utterance and that it is hence the other participant’s turn. The reason that this happens to a larger extent in the Experimental condition would then be that the emphasised backchannels more clearly signals understanding and encourages three-party interaction (see further the analysis of collaboration below). Considering the 13 L1–L2 interactions (7 in the Experimental condition and 6 in the Control), we first corroborate that the mean number of yielded speaker turns, for all timings, is much higher in the Experimental condition (M = 5.14) than in the Control (M = 0.83) also for this subset (M = 5.67, for all 9 Experimental interactions), and then find that the proportion of L2 speakers taking the turn is the same in the Experimental condition (M = 0.22) and in the Control (M = 0.2). Hence, the effect of the adaptive emphasized backchannels in the Experimental condition is that both L1 and L2 speakers take the turn more frequently than in the Control, but with the L1 speakers being substantially more active at taking the turn in both conditions.

To further investigate turn holding and yielding, a more detailed conversation analysis was performed of the sequences containing Turn-final and After-turn backchannels. The annotation protocol was that every backchannel occurrence was labeled in a separate text file with type (turn-final or after-turn), time in the video recording, speaker (left or right), turn-taking (holding, yielding, claiming), Furhat’s action (head nods, maintaining or shifting which interlocutor its head is turn towards, eye gaze, length of pause), the speaker’s verbal reaction (e.g., incremental, continuation, topic shift, pauses) and non-verbal reaction (e.g., head and body turns, gaze, laughing). We found that the generation of backchannels interconnects with the level of collaboration between the two human participants. Collaboration was analysed in terms of concrete practices in interaction, in particular (1) help-seeking, through asking the conversational partner directly or indirectly and with or without accompanying head-turns; (2) bodily orienting towards the partner and directing gaze toward the other, particularly during hesitations or problem of turn completion or continuation; (3) completing each other’s turns without any display of interruption; (4) conducting joint actions, e.g., laughing or sighing together, and finally, (5) generating backchannels on each other’s utterances and even giving (positive) feedback to each other, e.g., *yeah, right, correct, exactly* etc.

In general, collaboration firstly leads to more speaking activity for the human speakers and less pauses and silences in interaction, which results in a higher number of generated robot backchannels for participant pairs who collaborate more. Secondly, even if we found this interconnectedness between higher collaboration and higher number of backchannels in both the Experimental and Control condition, it was more prominent in the Experimental condition. Due to the relatively low number of interactions in each setting, it is not possible to determine with certainty that the higher level of collaboration in the Experimental condition is a result of the different robot backchannel strategy, but the findings do suggest that there is a direct relation between a higher frequency of backchannel-productions by the robot and increased collaboration. The four interactions that were assessed to be most collaborative had a higher number of turn-final and after-turn backchannels than the other sessions (averages of 11.5 *vs.* 4.8) and also a higher hold ratio for these backchannels (averages of 0.39 *vs.* 0.25).

We provide one example each of a highly collaborative and a highly non-collaborative interaction in Tables 2, 3. The transcripts have numbered lines for referential purposes and use L and R for the human subject on, respectively, the left and right seat and F for Furhat. Overlaps are shown with square brackets, pauses are shown in parentheses in seconds, and the degree symbol (° °) is used for quiet talk.

Table 2 illustrates how the human subjects collaborate to formulate cues for the robot. L turns to R and seeks confirmation by turning the torso and head to meet gaze (#01), and at times (#05 and #09) this is accompanied by direct questions, e.g., “*or?*”. They also help each other to continue or complete the turn. For instance, in #06, the robot turns to R to allocate the turn, and R initiates a contribution (#08), but soon yields the turn, which is immediately taken over by L (#09), who produce a clue, but also seeks confirmation from R who confirms it (#11). During this negotiation, the robot produces two backchannels (#10 and #13), which are followed by further collaboration between the human interlocutors (#15–16).

In contrast, in Table 3, we instead see one of the least collaborative pairs, as there are not much help-seeking, mutual gaze or any other verbal or embodied coordination for turn-taking to exhibit co-ordination. From R’s first utterances (#01–03) to L’s contribution (#14), approximately 20 s pass without any verbal or non-verbal exchange between the participants to display that they are engaging in a collaborative interaction. All interlocutors, including the robot, wait until somebody takes the floor (#3–14). During this time, participants show some signs of impatience with deep breath, click sound (“*tsk*”), smacking the lips, and drumming with their fingers on their legs. As the robot is set to respond to descriptions with guesses or backchannels, rather than taking own initiatives, this lack of collaboration also leads to less production of backchannels.

The collaboration between human subjects is dependent on the rapport that they form with each other, which in turn is influenced by socio-cultural factors, such as if the pair is matched in lingusitic level, gender, age and personality (extraversion) and how familiar, and hence comfortable, they are with interacting with a robot. The language proficiency did not affect the level of collaboration, which is also illustrated by the collaborative interaction in Table 2 between an L1 (left) and an L2 (right) speaker and the non-collaborative interaction between two L1 speakers in Table 3. As most interactions, including the two in the examples, were between one male and one female (5 interactions), with only two female-female and one each of male-male and male and non-disclosed, it was not possible to investigate gender effects on collaboration. Regarding age, the collaborating pairs were older (M = 35.4, SD = 10.8) than the less collaborating ones (M = 29.4, SD = 7.8) and had a slightly larger age difference within the pair (M = 14.3 *vs.* 10.4), potentially indicating age-related difference in social conventions for interaction. The collaborating pairs further self-assessed to be less extrovert than the less collaborating pairs (average pair sums M = 5.3, SD = 1.1 *vs.* M = 6.0, SD = 0.7). The reason that the less extrovert subjects collaborate more, which might seem counter-intuitive as extroversion is usually considered to promote inter-personal interaction, may be that the individuals in the more extrovert pairs were instead competing more to be dominant, which is also a trait of extroversion.

An additional factor that influences the rapport is the prior level of acquaintance between the subjects. The L1 and L2 speakers were recruited through separate systems and assigned to sessions independently to avoid that closer acquaintances were paired, but some pairs may nevertheless have had some familiarity with each other. Finally, we are interested also in if socio-cultural factors influence how the participants perceived the robot, which we explore in the next section.

### 4.3 Culturally-linked perception of robot backchannels

Table 4 summarises the post-session answers, showing that the robot was overall perceived as attentive and friendly, and in addition understanding in the Control condition. The robot’s interaction was assessed as balanced between the two speakers and the robot was rated as slightly more extrovert and human-like than a neutral level. There were no significant differences in the ratings between the Experiment and Control groups overall. Neither were there general significant differences in rating between L1 and L2 speakers in Control condition, between L1 speakers in Experimental setting and Control, and between L2 speakers in Experimental setting and Control. For L1 and L2 speakers in the Experimental setting, one significant (*p* = 0.02) difference was found, i.e., that the L2 speakers found that the robot’s interaction was slightly unbalanced towards them (M = 3.4), while the L1 speakers found that the robot’s interaction was balanced (M = 3.0). From an application point of view, this is interesting, since it could indicate that the L2 speakers became aware that the robot was trying to encourage their interaction, but that this was achieved without the L1 speakers feeling neglected.

**TABLE 4:** Post-session answers from the Control and Experimental group. Numbers are mean and standard deviation on a five-point Likert scale 1–5.

	Understanding	Attentive	Friendly	Extrovert	Balance	Human-like
Control	3.8 ± 0.7	4.1 ± 0.8	3.8 ± 1.0	3.1 ± 0.5	3.0 ± 0.5	3.6 ± 1.0
Experiment	3.3 ± 1.1	4.0 ± 0.9	4.0 ± 0.9	3.5 ± 0.9	3.2 ± 0.4	3.0 ± 1.3
All	3.6 ± 1.0	4.1 ± 0.8	3.9 ± 0.8	3.3 ± 0.8	3.1 ± 0.5	3.2 ± 1.2

**FIGURE 5:**
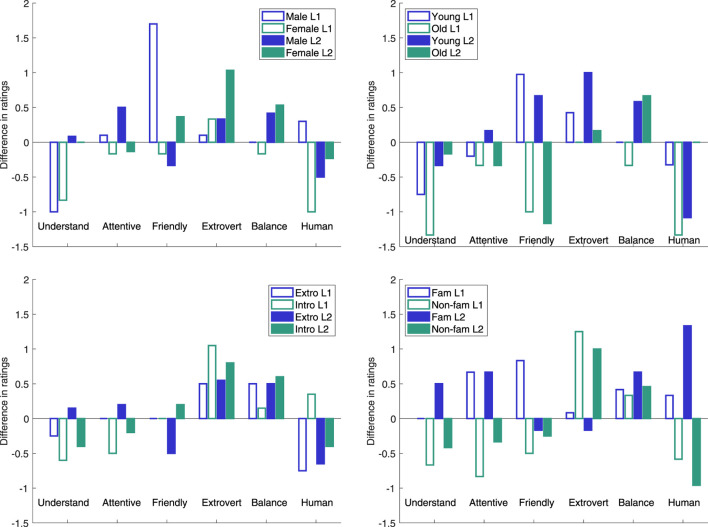
Differences in five point Likert scale ratings between Experimental and Control conditions depending L1 or L2 speaker, in combination with Upper left: male or female, Upper right: younger or older than the mean age in the group, Lower left: more (Extro) or less (Intro) extrovert than the mean level in the group or Lower right: more (Fam) or less (Non-fam) familiar with robots than the group mean.

Figure 5 illustrates the per group difference in mean ratings between the Experimental and the Control conditions for different socio-cultural factors, and the most important differences between the ratings in the two conditions were:For *gender*, male L1 speakers rated the robot’s friendliness substantially higher, female L2 speakers the extroversion higher, female L1 speakers the human-likeness lower and both male and female L1 speakers the understanding lower. Female subjects overall rated the robot significantly (*p* = 0.01) higher regarding attentiveness (M = 4.4) than the male did (M = 3.7), but otherwise there were no statistical differences in the ratings from the two genders.For *age*, older speakers rated the robot’s friendliness substantially lower in the Experimental condition, whereas the younger speakers rated it higher, and the difference between the age groups was significant (*p* = 0.02) for the Experimental condition. The older L1 speakers in addition rated the understanding and the human-likeness lower; in agreement with younger L2 speakers in the latter case. The younger subjects overall rated the robot’s extroversion significantly higher (*p* = 0.03) in the Experimental condition (M = 4.3) than in the Control (M = 3.6), whereas the older subject rated its friendliness significantly (*p* = 0.04) lower in the Experimental condition (M = 3.2) than in the Control (M = 4.2).For *extroversion*, the introvert speakers rated the robot’s understanding and attentiveness lower and extroversion higher, regardless of condition. For the Experimental condition, extrovert subjects also rated the robot’s extroversion higher (but less so than the introvert) but the human-likeness and understanding lower. The difference for understanding (M = 2.6 *vs.* M = 3.9) was significantly (*p* = 0.01) lower than in the Control and this rating was also significantly (*p* = 0.04) lower than from the introvert subjects (M = 3.7). For *familiarity with robots*, the subjects with below-mean familiarity rated the robot’s understanding, attentiveness, friendliness and human-likeness lower and extroversion higher in the Experimental condition, often in contrast to the subjects with above-mean familiarity, who had the opposite view for understanding (L2 subjects), attentiveness and human-likeness (both L1 and L2 subjects) and friendliness (L1 subjects). The rating of human-likeness by the subjects with below-mean familiarity with robots was significantly (*p* = 0.01) lower in the Experimental condition than in the Control (M = 2.8 *vs.* M = 4.0). The latter rating was further significantly (*p* = 0.01) higher than the rating from the subjects with above-mean familiarity (M = 2.7).

The set of L1s was too heterogeneous to make any statistical analysis of post-session survey differences between L1s or L1 language groups. Further, we initially hypothesised that educational level, as a proxy for sociolect, could influence the perception of robot backchannels, but since all recruited subjects were at university undergraduate or graduate level, the group was too homogeneous to explore this.

A multilinear regression analysis was performed for each of the post-survey categories to investigate how experimental condition, gender, L1/L2 speaker and age plus their interactions influenced the ratings. The only statistically significant (*p* = 0.01) relationship found was for the rating of the robot’s friendliness, for which the male subjects’ rating increased with age, regardless of condition and regardless of if they were L1 and L2, and the female subjects’ rating decreased with age in the Experimental condition for both L1 and L2 subjects, while it was close to constant in the Control.

To summarise the post-session survey, we hence found that H3 is confirmed. The rating of the robot’s*Understanding* was affected by the subject’s extroversion (presumably since extrovert subjects were more active as speakers, making the robot’s task of indicating understanding harder). *Attentiveness* was affected by gender, in line with earlier findings (Mulac et al., 1998). *Friendliness* was affected by age and the combination of age and gender, as older [female] subjects found the robot to be less friendly when its backchannel strategy differed between the two speakers, possibly based on age-related expectations on polite listener behaviour. *Extroversion* depended on the condition, as the robot was perceived as more extrovert when it was set to encourage the least speaking participant and therefore had more expressive vocal and visual backchannels. *Balancing behaviour* differed between L1 and L2 speakers in the Experimental condition, as already discussed above. *Human-likeness* in the Control condition was influenced by how familiar the subjects were with interacting with robots, which is in line with previous findings on the novelty effect, which signifies that first-time users are initially more positive towards new technology.

## 5 Discussion

### 5.1 Limitations

The experiment is of a short interaction with a small number of L2 subjects per L1, which makes it difficult to draw general conclusions regarding how listeners of that particular L1 respond to and perceive robot backchannels. It would therefore be valuable to carry out a similar experiment with larger groups of subjects from the same L1, who would ideally also interact with the robot for a longer duration.

Even when grouping all L2 speakers together for comparisons between Experimental and Control conditions for other socio-cultural factors, the number of subjects per group is quite low and, for many dimensions, the observed differences were not statistically significant, and can hence only provide insights into potential effects that should be investigated for a larger subject group. The experiment is moreover set up as a between-subject comparison, and other personal characteristics than the ones investigated in the experiment may hence have influenced the results.

Regarding the influence of L1, we have actually not investigated whether L1 and L2 speakers react differently to a similar robot backchannel strategy, since the experiment focused on providing the less active speakers—which was most often the L2 subjects—with relatively more and more expressive feedback than the more active—mostly L1—speakers. A focused follow-up, in which less active L1 speakers in L1–L1 interactions receive the same type of additional backchannels as the L2 speakers in this study, would be required to study if L2 speakers respond differently to the *same* robot backchannels, but this was outside the scope of the present study.

Speaking time is one measure of how proficient a speaker is, and as shown in Figure 2, the L1 speakers did indeed overall speak more than the L2 speakers in the Control condition (and less so in the Experimental condition). With the aim of encouraging the L2 learners to practice speaking more in this setting, speaking time is thus a simple measure to identify which speaker to encourage. However, speaking time disregards the linguistic content, as well as the speaking rate, of the utterances, which can potentially bias the backchanneling behaviour, since native speakers probably speak faster and may be able to produce shorter, more concise utterances to convey the same message as an L2 speaker who struggle to find the exact words. We did not generally observe such effects in our interaction analysis, but it needs to be considered for less structured interactions, and we acknowledge that the results may be specific for this type of game set-up.

Similarly, speaking time accumulated over the entire session is a suitable for this type of separate (one per game word) and short (60 s per game word) interactions, whereas another measure would be required for longer and less structured conversations, in which different speakers may dominate sequentially. For the latter case, it may be more appropriate to estimate the dominating speaker based on a sliding time window.

It should finally be noted that the nature of backchannels is such that they can only be delivered towards someone who is speaking. This signifies that the absolute number of backchannels will be low for subjects who speak very little, even in the Experimental condition when the robot provided relatively more backchannels towards the least speaking subject (two subjects in the Experimental and two in the Control condition received no or one backchannel each). Moreover, the adaptive backchannel strategy in the Experimental condition has the side effect that if one of the speakers is very inactive, then the more active speaker will also receive few backchannels, since the robot aims to display a higher relative proportion of backchannels towards the other speaker (three speakers in the Experimental condition therefore received no or only one backchannel because the other participant was speaking so little). An improved adaptive backchanneling strategy, when the time limit is less strict, would be to ensure a minimum level of backchannels towards an active speaker, even if the other participant is silent.

### 5.2 Future work: Implications for culturally aware robots

This study has investigated how users of different socio-cultural backgrounds perceive and react to robot backchannels as a first step towards creating culturally aware robots. The next step would be to use the information to adapt the robot’s behaviour and interaction to suit the present users. Based on the results above, we suggest that the robot’s backchannels may be adapted in the following ways:

#### 5.2.1 Function of robot backchannels

The experiment showed that Participation-adjustment, i.e., to display relatively more backchannels towards the least-speaking participant, contribute to a more balanced interaction, but is—at least with the current formulation and timing—more effective for male, younger and extrovert speakers, compared to female, older and introvert speakers. The formulation, timing and frequency of backchannels may therefore have to differ for different socio-cultural groups in order for them to have the same function.

Further, the conversation analysis showed that the social rapport between the participants influences how the robot backchannels are perceived, as speakers in collaborative pairs are more likely to hold the turn after a turn-final or after-turn backchannel than non-collaborative speakers. The intended function of the robot back channels—e.g., to promote collaboration or to specifically encourage the current speaker to continue—should hence influence timing, frequency and formulation of backchannels.

#### 5.2.2 Timing and frequency of robot backchannels

Gender and/or age influence expectations on the interplay between interlocutors in a conversation, regarding what constitutes a friendly, attentive listening behaviour. The backchannel strategy in the present study was the same towards all subjects in the same condition, but observations from the reactions (in terms of speaking time ratios, multimodal responses and post-session ratings) suggest that female and older subjects may expect more and more explicit backchannels that are also more equally distributed between interlocutors in order to perceive the robot to be polite and encouraging speaking activity.

In addition to the above differences in perceiving backchannels, it should be noted that previous studies have shown that there are gender differences in producing them, and the robot backchannels may need to be adapted if a male voice is used for the robot. Similarly, the Furhat robot used in this study is more anthropomorphic in appearance and interaction signals than most other commercially available robots. It remains to be shown if similar effects would be obtained also with less anthopomorphic robots.

#### 5.2.3 Formulation of robot backchannels

Introvert speakers should receive more explicit verbal backchannels, formulated to clearly encourage them to continue, when this is the robot’s intention. This may in particular be required for subjects with low familiarity with robots, as they seem to perceive robot backchannels differently than more experienced subjects. It should also be considered that there may differences in the subjects’ responses to robot backchannels depending on if they are formulated to encourage the less active speaker (as in this study) or to restrain the more active speaker (in order to provide the peer with more opportunities to speak).

#### 5.2.4 Combined timing, formulation and function of backchannel

The multimodal analysis further suggests that the timing and formulation of the backchannels influence how the interlocutors perceive their function, as Turn-final and After-turn backchannels often encouraged speakers to yield the turn rather than to hold it. Backchannel formulation should hence correspond to its intended function for the timing when it is issued.

The EMCA analysis identified a number of age-gender-extroversion related differences, which could be used if a rule-based backchannel strategy is employed, but a data-driven approach may hold larger potentials to learn formulation, timing and frequency of backchannels for different interlocutor categories (such as age, gender and extroversion level) from a multimodal spoken interaction database, e.g., Spontal (Edlund et al., 2010).

## 6 Conclusion

Starting from the findings in previous research that backchannel production and perception differ between different socio-cultural groups and that robot backchannels may shape HRI, we have shown, in a multi-party experiment, that the robot’s backchanneling strategy has a substantial impact on how two speakers distribute their speaking time (H1) and that both the distribution of speaking time (H2) and the perception (H3) of the robot are influenced by several socio-cultural factors. These influenced the effectiveness of the encouraging backchannels and we have therefore proposed a number of adjustments to different socio-cultural factors that should be made if a rule-based backchanneling strategy is used. The proposed changes should be evaluated in future user studies, but may also be calibrated with and compared to observed backchannel strategies in existing multimodal databases of human-human interaction. Backchannels play a very important role in natural spoken interactions between humans and based on findings such as the ones from this study, robots may be endowed with backchannel capabilities that make them more culturally aware.

## Data Availability

The datasets presented in this article are not readily available because the datasets generated for this study are not public, due to privacy limitations to comply with the consent form signed by the subjects. Request to access anonymised parts of the datasets should be directed to engwall@kth.se.

## Footnotes

1
https://www.cs.utep.edu/nigel/bc/common-bcs.html, accessed 2022/04/07.

2 Available in the public GitHub repository https://github.com/ronaldcumbal/medandraord.
